# Patient expectations and levels of satisfaction in chiropractic treatment for lumbar radiculopathy. A mixed methods study

**DOI:** 10.1186/s12998-023-00486-0

**Published:** 2023-05-19

**Authors:** Rikke Krüger Jensen, Sille Lillesø, Jack Starche Jensen, Mette Jensen Stochkendahl

**Affiliations:** 1 Department of Sport Science and Clinical Biomechanics, University of Southern Denmark, Campusvej 55, Odense M, 5230 Denmark; 2 Chiropractic Knowledge Hub, Campusvej 55, Odense M, 5230 Denmark

**Keywords:** Radiculopathy, Standardised care package, Chiropractic, Patient satisfaction

## Abstract

**Background:**

Chiropractic patients are generally satisfied with the care received. It is unclear if this also applies to Danish patients with lumbar radiculopathy included in a standardised chiropractic care package (SCCP). This study aimed to investigate patient satisfaction and explore perspectives on the SCCP for lumbar radiculopathy.

**Methods:**

An explanatory sequential mixed methods design with three separate phases was used. Phase one was a quantitative analysis based on a survey in a prospective cohort of patients with lumbar radiculopathy in an SCCP from 2018 to 2020. Patients rated their satisfaction with the examination, information, treatment effect, and overall management of their problem on a 0–10 scale. In phase two, six semi-structured interviews conducted in 2021 were used to gain further explanatory insights into the findings from phase one. Data were analysed using systematic text condensation. In phase three, the quantitative and qualitative data were merged in a narrative joint display to obtain a deeper understanding of the overall results.

**Results:**

Of 303 eligible patients, 238 responded to the survey. Of these, 80–90% were very satisfied (≥ 8) when asked about the examination, information, and overall management, whereas 50% were very satisfied with the treatment effect. The qualitative analysis led to the emergence of four themes: ‘Understanding the standardised care packages’, ‘Expectations regarding consultation and treatment effect’, ‘Information about diagnosis and prognosis’, and ‘Interdisciplinary collaboration’. The joint display analysis showed that high patient satisfaction with the examination could be explained by the patients’ feeling of being carefully and thoroughly examined by the chiropractor and by referrals to MRI. Advice and information given to patients on variations in symptoms and the expected prognosis were considered reassuring. Satisfaction with the chiropractor’s coordination of care and with referral to other healthcare professionals was explained by the patients’ positive experiences of coordinated care and their sense of alleviated responsibility.

**Conclusion:**

Overall, patients were satisfied with the SCCP for lumbar radiculopathy. From a patient’s perspective, the consultation should include a thorough examination and a focus on communication and information relating to symptoms and prognosis, while expectations regarding the content and efficacy of the treatment should be addressed and aligned.

**Supplementary Information:**

The online version contains supplementary material available at 10.1186/s12998-023-00486-0.

## Background

Information on patient-reported outcomes of effect and patient experienced quality of care is central to evaluating healthcare processes [1] and patient satisfaction is a proposed patient-focused outcome for clinical practice [2]. Patient satisfaction is generally recognised as a multidimensional factor related to several aspects of the patient’s experience throughout consultation and treatment and assessed by subjectively evaluating the experience with the healthcare service and the clinician [3–5]. In patients with low back pain (LBP), views on the quality of care and of healthcare programmes have traditionally been measured by patient satisfaction [6] which is recommended as one of the core outcome measures in back pain research [7]. Individual treatment success on goal achievement scores for patients with LBP has been shown to be more strongly associated with patient satisfaction than with outcomes in pain and physical function [8]. When patients with LBP consult a clinician, they expect an explanation of the cause of their pain, a reduction in the level of pain, and improvements in their ability to perform various tasks [9]. A systematic scoping review [10] identified that patients with LBP valued good communication skills in their healthcare provider, including open, patient-centred communication, respectful listening, empathy, understanding and shared decision-making. They wanted individualised treatment and continuity of care, information about the cause of their LBP, and legitimisation of their symptoms. They perceived lengthy waiting times, costs, and obstacles to treatment as being challenges to the management of LBP.

Most healthcare providers in primary care practice settings work in health systems regulated by government organisations and professional bodies. Organisational regulation enables national health systems to shape the behaviour of healthcare providers, allowing national health authorities to control the quality and safety of care offered by health professionals and to regulate the market in healthcare services [11]. Regulations can include disease management programmes such as healthcare packages, ensuring that clinicians deliver services in agreement with current national guidelines on clinical excellence, and they are often designed to ensure quality and consistency of patient management. Healthcare packages also serve the purpose of directing resources to specific areas of health service delivery with high priority, such as disease management programmes for persistent musculoskeletal pain conditions. However, implementing national health initiatives in clinical practice can be challenging, as not only resources but also clinicians’ attitudes and behaviours play a role in implementation success [12, 13].

In Denmark, chiropractors are regulated by the National Health Care Authorities, and the collective agreement includes increased partial reimbursement for standardised care packages for lumbar radiculopathy [14]. Most patients with radiculopathy are managed in primary care by general practitioners (GPs), physiotherapists, and chiropractors [15] and constitute a disease group associated with increased pain, disability, poor quality of life, and increased use of health resources compared with LBP without radiculopathy [16]. The care package was developed to help support professionals and describes a management structure and the logistics of a patient care pathway with which Danish chiropractors are obligated to comply. The programme’s content adheres to Danish national clinical guidelines, which require that the patient be apprised of their condition and prognosis, provided with information, guidance and advice on staying active, and offered supervised exercise and manual therapies as needed [17].

Although chiropractic patients generally report high levels of satisfaction with the care they receive [18, 19], it is unclear if this reflects on the structured, standardised care programme concerning patients with radiculopathy. Therefore, this study aimed to describe patient satisfaction and explore patient perspectives on the Danish SCCP for lumbar radiculopathy.

## Methods

### Design

**Fig. 1:**
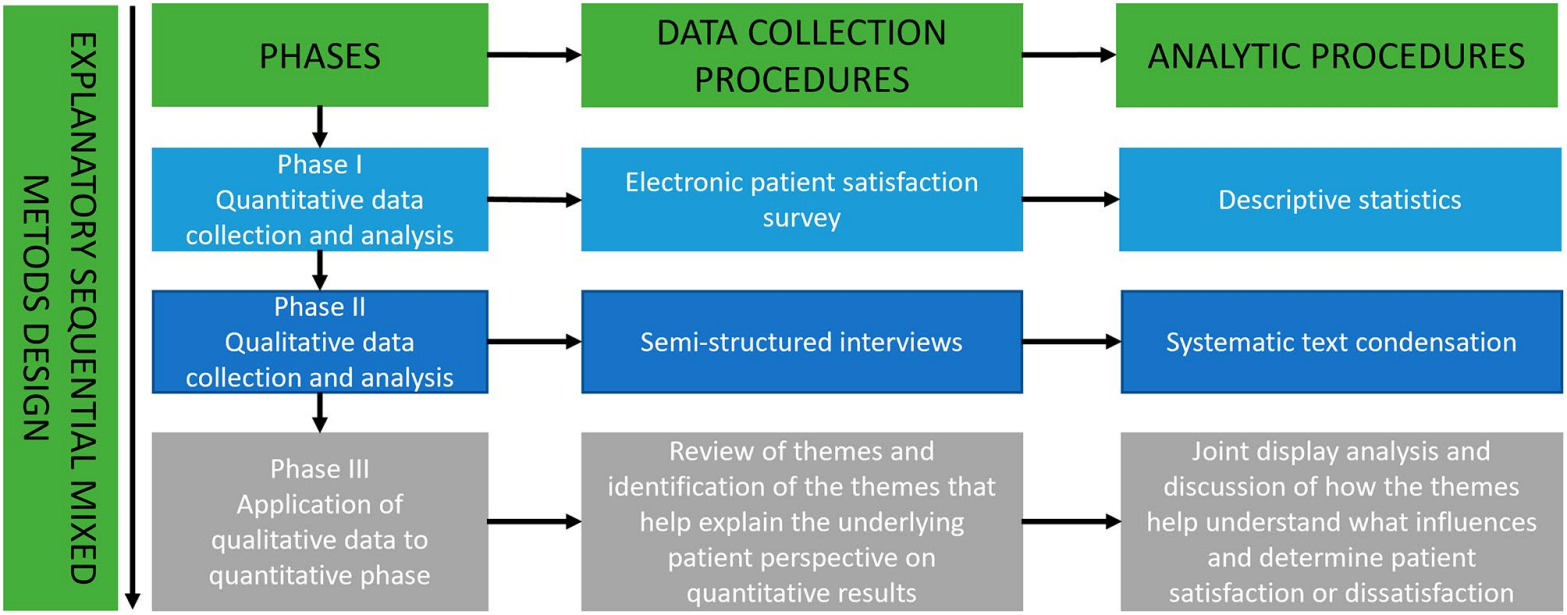
Overall flow of the design *Procedural diagram of the explanatory sequential mixed methods design showing the procedures and products for each phase horizontally*

This study used an explanatory sequential mixed method design consisting of quantitative and qualitative data collection elements. In this design, quantitative data collection is used to gain an overview of the subject matter. Next, qualitative data collection is used to explain the results of the quantitative data. The rationale for using this approach is that qualitative data can reveal emergent themes and interesting quotes that can be used to explain quantitative findings in the words of participants and gain multiple perspectives on the research question, which will enrich the overall results and capture nuances [20]. Prior to conducting this study, we expected high satisfaction levels based on previous studies [18, 19]. The qualitative results would then help explain which factors may be drivers of positive experiences and whether there were elements that patients felt were missing or would have liked. Explanation of low levels of satisfaction would improve the understanding of what lies behind negative answers. Either way, results could potentially improve healthcare services in the future. The overall flow of the design is shown in a procedural diagram (Fig. 1). The study was reported following the ‘Good Reporting of A Mixed Methods Study’ (GRAMMS) framework [21].

### Phase I: quantitative data

#### Study population and data collection

The study population was made up of patients with lumbar radiculopathy enrolled in a Standardised Chiropractic Care Package (SCCP) at a Danish chiropractic clinic. The SCCPs are part of a collective agreement negotiated by the Danish Chiropractic Association and Danish Health Authorities [14] and specify a higher proportion of reimbursement of the patient fee (40–60%) for patients in the SCCP compared to regular fees (9–18%). The SCCP does not stipulate a particular treatment or type of care but a management structure and a logistical system of patient care, including pre-scheduled follow-up sessions at 2, 4 and 8 weeks. Additional consultations may be scheduled as needed. The pre-scheduled consultations must include monitoring the progression of symptoms based on the patient’s history and the clinical examination, reassessment of the treatment plan (including referral to imaging or surgical evaluation) based on the patient’s status, and providing standardised written information to the patient’s GP.

All chiropractic clinics with a collective agreement (n = 153) located in one of three Danish regions (Region of Southern Denmark, Zealand Region, and Capital Region Denmark) received an invitation by e-mail to participate in the study. If clinics did not respond to the e-mail (n = 143), they were contacted by phone ten days later.

Consecutive patients with lumbar radiculopathy of less than three months duration who started an SCCP from April 2018 to April 2020 were invited to participate in the project. Patients were included if they were 18 years or older and could speak, read and understand Danish. The diagnostic criteria of radiculopathy were based on an overall assessment of the case history and clinical findings, as there is no specific test for radiculopathy but a combination of positive findings on examination increases the likelihood [15]. The diagnostic criteria for lumbar radiculopathy defined in the collective agreement [14] included: leg pain more intense than LBP, positive nerve stretch test, and possibly one or more signs of neurological symptoms such as sensory disturbances, lack of reflexes, or loss of muscle strength. Patients who met the above criteria and, in addition, had physical limitations due to symptoms that had been present for less than three months were included in the SCCP.

If the patients fulfilled the inclusion criteria, the chiropractor or secretary at the clinic would briefly inform them about the project, provide written information, and enter contact details in the electronic online system Research Electronic Data Capture (REDCap). Patients were then contacted by e-mail, which would include a link to a comprehensive electronic questionnaire. Non-responders received a reminder by e-mail after three days. Participants who did not respond to the reminder were contacted by phone by a research assistant who checked for a valid e-mail address and resent the questionnaire after receiving permission.

#### Variables of interest

The following data were collected at baseline: age, sex, level of education, whether the patient had visited other healthcare providers for the current problem, current use of pain medication, duration of back and leg pain (days), back and leg pain intensity measured on an 11-point numerical rating scale (NRS) [22], and physical function measured by the Oswestry Disability Index (ODI) on a 0-100 scale [23, 24].

Patient satisfaction was explored using four questions and measured on a scale from 0 ‘Very dissatisfied’ to 10 ‘Very satisfied’: “How satisfied are you with the examination performed by the chiropractor?”, “How satisfied are you with the information you received about your problems?”, “Overall, how satisfied are you with the chiropractor’s management of your problems?” and “How satisfied are you with the effect of the treatment you received for your problems?”.

#### Analyses and data management

Baseline characteristics were presented as means with standard deviations (SD), medians with IQR (depending on the data distribution) or frequencies and proportions. Patient satisfaction was analysed with descriptive statistics and visualised with graphics. When merging with the qualitative data and in the presentations, we dichotomised satisfaction into ‘Very satisfied’ (≥ 8 on the 0–10 scale) and ‘Less satisfied (< 8)’. The cut-point was arbitrarily chosen to identify the top 30% of the scale as very satisfied. Analyses were performed using STATA 17 (Stata Corp, College Station, Texas, USA).

### Phase II: qualitative data

#### Study population

The inclusion criteria for patients in the qualitative phase of the study were similar to the quantitative phase. To ensure that patients had experience with the programme and to minimise recall bias, the patient had to be a minimum of four weeks into the SCCP or should have completed the programme within the last month, which would be a maximum of three months from baseline. Patients recruited for the interviews were not part of the survey cohort due to the time difference between data collection for the two phases of the project and therefore did not complete the survey.

#### Recruitment

All chiropractic clinics in Denmark were identified using a list of clinics from a public website hosted by the Danish Health Authorities [25]. We targeted large chiropractic clinics (≥ 3 clinicians), as they were expected to have a higher patient flow than smaller clinics, potentially shortening the recruitment period. Two clinics from each of the five geographical regions in Denmark were selected using an online random sampling tool [26]. The randomly selected clinics were contacted by phone, and chiropractors were invited to act as gatekeepers in the recruitment of patients. If a clinic declined to participate, a new clinic was randomly selected from the remaining clinics in that same region. Written information, including consent to participate and practical instructions, was sent by e-mail to the clinic. Gatekeepers were instructed to obtain consent from eligible patients and collect contact information.

Gatekeepers were instructed to include all eligible patients and no measures were taken to ensure variability in satisfaction across the sample. The method used was thus convenience sampling due to the availability of eligible patients and the need for expedited data collection. Patients identified by gatekeepers were contacted directly by two of the authors (JSJ and SL), who assessed inclusion criteria, provided project information, and made an appointment for an interview.

#### Data collection

An interview guide was developed based on the content and results of the quantitative data. The general topics in the interview guide were patient satisfaction with the (a) examination, (b) information, (c) treatment effect, and (d) overall management. It also included an exploration of knowledge, expectations and experience with the SCCP. The interview guide included open-ended questions such as, ‘How did you find the examination you received at the chiropractor?’ ‘What do you think about the structure of the treatment course?’ to allow the patients to share their experiences and express their views. The interviewers focused on the follow-up questions and gave time for reflection. The complete interview guide is provided in Additional file 1. Information on age, sex and level of education was also collected. Prior to data collection, the interview guide was pilot tested on one patient and revised based on the feedback.

One of two authors (JSJ and SL) conducted the semi-structured interviews between February 15th, 2021 and March 26th, 2021. The interviewers were master’s students in their last year, and the project was part of their master’s thesis. In line with the informational power approach [27], sample size estimation was based on the specificity of the study aim (here: narrow), the specificity of the sample (here: dense), the use of established theory (here: applied), the quality of the dialogue (here: expected to be weak due to the limited experience of the interviewers), and the analytical strategy (here: cross-case). The judgements made using this framework lead to smaller sample size requirements and a sample size of 10 participants would be considered sufficient. Due to the COVID-19 pandemic, the interviews were conducted online via the communication service Zoom (Zoom Video Communications, Inc. Version: 5.4.7 (59780.1220)) and recorded as audio files.

#### Analyses and data management

The author who conducted the interview transcribed the interview using the Microsoft Word programme (for Mac, version 16.47, Microsoft). The transcript was then uploaded to the data analysis software NVivo 12 (QSR International, MAC version 12.3.0 (3508)), also used for the data analysis. The transcript quality was inspected by the author who had not conducted the interview, and revisions were made as necessary. The audio files were continuously processed and analysed using ‘Systematic Text Condensation’ (STC) [28]. Based on SCT, the analysis was performed step by step to uncover themes presented in the interviews [28].

**Fig. 2:**
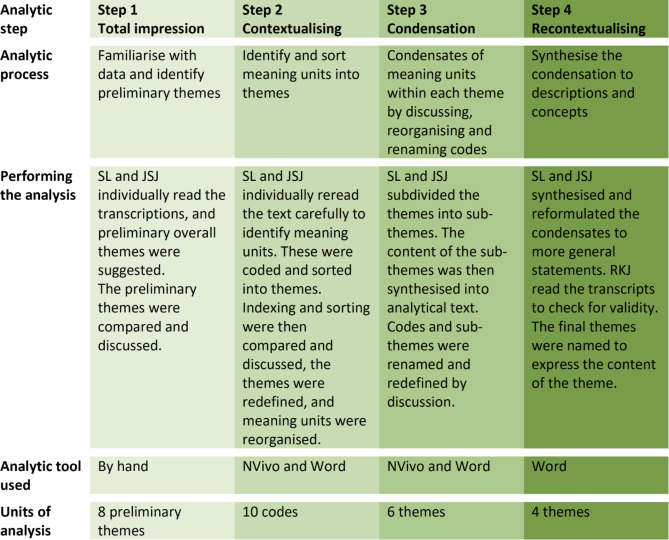
The analytic process

The SCT analysis involved the following steps. First, the transcriptions were read individually in a ‘naïve’ way to become familiar with the data and gain an initial understanding of the patient’s experience and their views on standardised care. Preliminary overall themes were suggested. Secondly, meaning units were identified, coded and sorted individually into the preliminary themes by two authors. Indexing and sorting were then compared and discussed between the two authors. The themes were redefined or identified, and the meaning units were reorganised to reach a consensus. Thirdly, themes were subdivided into sub-themes identifying different patient perspectives within each theme. The content of the sub-themes was then synthesised by discussing, renaming and redefining the codes. Finally, the condensates were synthesised and reformulated to more general statements, and the final themes were named to express the theme’s content. The analytic process is further presented in Fig. 2.

### Phase III: merging quantitative and qualitative data

The qualitative findings expanded the quantitative results by providing further insight into patients’ perspectives of the SCCP. The integration was visualised as a narrative side-by-side joint display created at the end of the data collection process of the qualitative phase to synthesise the quantitative survey components and the qualitative results [29]. First, the dichotomised quantitative data were chosen as a point of comparison in the joint display. The contributing themes of the qualitative phase were then mapped against the quantitative results, and finally, illustrative key quotes were linked to the themes.

## Results

### Phase I: quantitative results

Of the 153 clinics invited, 49 (32%) agreed to participate, and 31 clinics (65 clinicians) had enrolled one or more patients by the end of the study.

A total of 303 patients with lumbar radiculopathy were invited to participate in the survey, of which 238 (79%) were included in the final study population. Reasons for exclusion were doublets (n = 1), mail errors (n = 3), declined consent (n = 3), no age or sex information (n = 1), withdrawal due to COVID-19 (n = 1), and 56 did not fill out the baseline questionnaire.

The mean age of the patients was 47 years (SD 14), and 52% were female. Almost half had visited another care provider for their current problem, most often their GP, and around 80% were currently taking pain medication. (Table 1)

**Table 1:** Baseline characteristics of patients with lumbar radiculopathy receiving a standardised care package

	Missing (%)	Total
n		238
Age, mean (SD; full range) (n = 237)	1 (0.4)	47.4 (14.3; 18–84)
Sex, female; n (%)	0 (0)	124 (52.1)
Most formal education, n (%)	22 (9.2)	
− Primary school 8 to 10 grade		20 (9.3)
− High school		21 (9.7)
− Vocational education		76 (35.2)
− Academic, maximum 4 years		68 (31.5)
− Academic, > 4 years		31 (14.4)
Seen by another healthcare provider for the current problem (yes), n (%)	9 (3.8)	102 (44.5)
− General practitioner, n (%)		79 (77.5)
− Chiropractor, n (%)		25 (24.5)
− Physiotherapist, n (%)		57 (55.9)
Currently taking pain medication for back or leg pain, n (%)	9 (3.8)	
− No		50 (21.8)
− Yes, prescription medicine		108 (47.2)
− Yes, over-the-counter medicine		71 (31.0)
> 30 days with LBP during the previous year, n (%)	14 (5.9)	99 (44.2)
> 30 days with leg pain during the previous year, n (%)	15 (6.3)	59 (26.5)
Low back pain, mean (SD)	1 (0.4)	6.2 (2.7)
Leg pain, mean (SD)	0 (0.0)	7.0 (2.4)
Oswestry Disability Index, mean (SD)	15 (6.3)	40.0 (18.9)
Satisfaction with the examination, median (IQR)	11 (4.6)	10.0 (2.0)
Satisfaction with the information, median (IQR)	14 (5.9)	9.0 (2.0)
Satisfaction with the effect of the treatment, median (IQR)	18 (7.6)	8.0 (3.0)
Satisfaction with the overall management, median (IQR)	11 (4.6)	9.0 (2.0)

IQR: interquartile range; SD: standard deviation

**Fig. 3:**
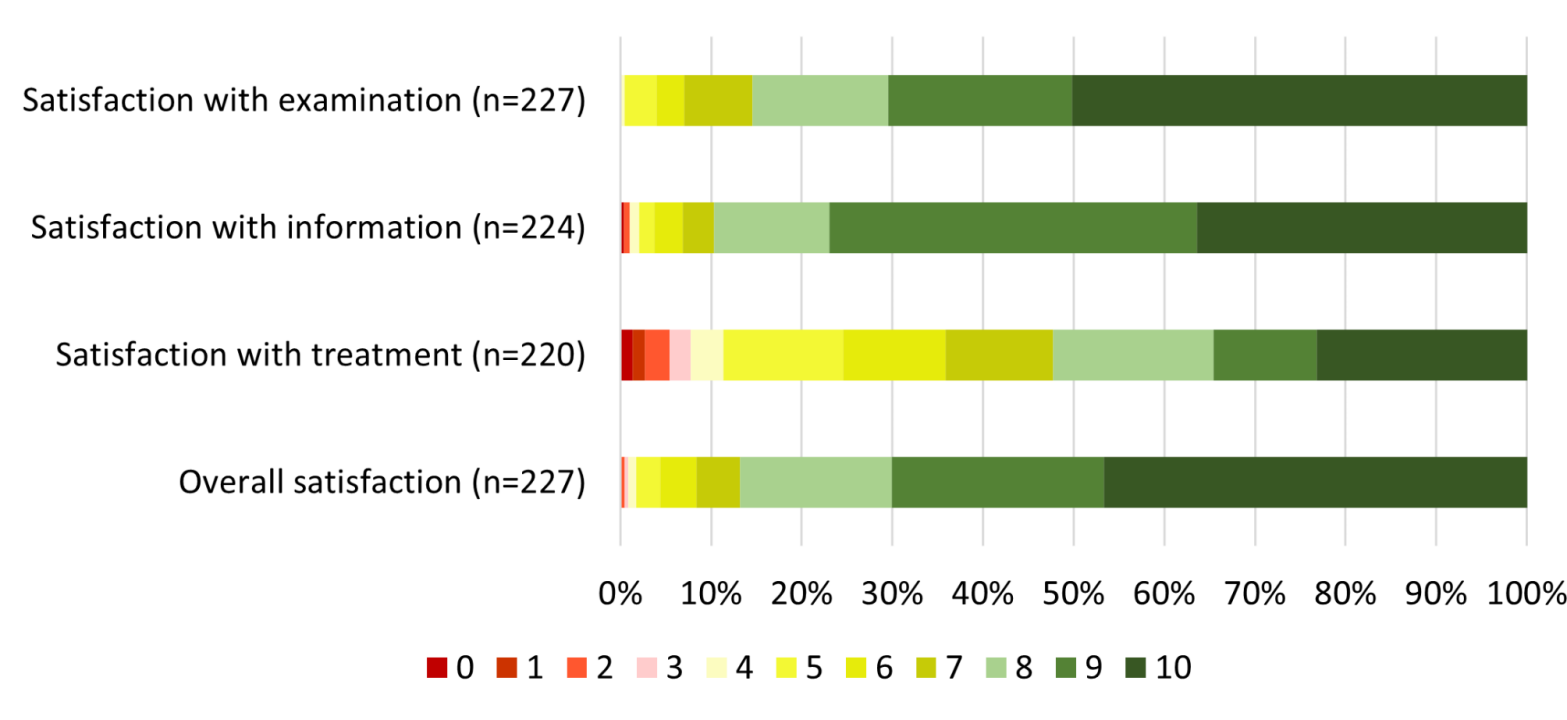
Patient satisfaction with the examination, information, treatment effect, and the overall management of problems. *Scale 0-10 (0=very dissatisfied; 10=very satisfied)*

Patient satisfaction is presented with graphics in Fig. 3. Most patients were ‘Very satisfied’ (≥ 8 on the 0–10 scale) with the examination (86%), information (87%), and overall management (87%), while 52% were satisfied with the treatment effect.

### Phase II: qualitative results

A total of 14 clinics were contacted, of which two declined to participate, two did not respond, and ten agreed to recruit patients. Finally, five clinics recruited patients for six semi-structured interviews. Data collection was stopped after six interviews, as time did not allow for further enrolment of patients and preliminary results suggested that the data had sufficient information power to contribute with new knowledge. It was not possible to include patients from The North Denmark Region, the smallest of the five Danish regions as regards population, as no chiropractors were prepared to act as gatekeepers. The duration of the interviews was between 20 and 43 min. Characteristics of the patients are shown in Table 2.

**Table 2:** Characteristics of informants

Patient ID	Age in years	Sex	Level of education	The geographical region in Denmark
1	39	Male	Vocational education	Region Zealand
2	53	Female	Academic, maximum 4 years	The Region of Southern Denmark
3	55	Female	Academic, > 4 years	The Capital Region of Denmark
4	44	Male	Vocational education	The Region of Southern Denmark
5	53	Male	Vocational education	Central Denmark Region
6	26	Female	Academic, maximum 4 years	Central Denmark Region

Categorisation and analysis of the qualitative data led to the emergence of four themes described below: ‘Understanding of SCCP’, ‘Expectations regarding consultation and treatment effect’, ‘Information about diagnosis and prognosis’ and ‘Interdisciplinary collaboration’.

#### Understanding of SCCP

To most patients, it was unclear who was responsible for the organisation and referrals to the SCCP and what services were included in the programme. They were aware of the pre-scheduled follow-up sessions, which they considered reassuring on the one hand but, on the other, felt were inflexible. They were conscious of the increased partial reimbursement of treatment costs for patients allocated to the SCCP, which would decrease their out-of-pocket expenses.

#### Expectations regarding consultation and treatment effect

Two patients who had not been referred for MRI were disappointed with that decision. They felt it was important to get an MRI early in the course of their treatment as it would provide them with certainty and confidence by confirming the diagnosis. Two patients who had been referred for MRI confirmed that notion, as they explained that it had provided them with a better understanding of their condition. One patient explained how the diagnosis confirmed by MRI gave the chiropractor confidence and that this confidence was reflected in the patient. Patients emphasised that it was important for the chiropractor to deliver a comprehensive examination and spend time on the consultation, for otherwise it might feel like neglect of the patient.

Patients expected the consultations in the SCCP to include manual treatment, exercises and professional guidance in rehabilitation. If exercises were not provided, some patients expressed disappointment and dissatisfaction as they expected a more rapid improvement with additional treatment modalities such as exercises. One patient instructed in exercises felt the ‘symptom-guided approach’ was a heavy responsibility that made him feel less confident. Almost all patients explained that they had consulted the chiropractor in the expectation of a rapid decrease in symptoms that was expected to be permanent.

#### Information about diagnosis and prognosis

Patients valued detailed information and explanations being given about their pain condition. Specifically, they appreciated the chiropractor informing them about the prognosis, treatment plan and expected duration of symptoms. Patients perceived the provision of this kind of information as a reassuring activity that made them feel involved in their treatment. However, one patient received information about staying active that did not fit with the patient’s perceptions and led to anxiety, concern and distrust in the chiropractor.

Some patients explicitly mentioned using posters, drawings or anatomic models to support the explanations as enhancing their understanding of their condition. Also, the distribution of written information about the SCCP was appreciated, as some patients felt that there was much information to take in at the first consultation and that revisiting the information later was helpful for understanding the condition and the structure of the care package. When sufficient information was not provided, pain flair-ups made the patients anxious, as they did not know what to expect or what was considered a normal variation in pain intensity.

#### Interdisciplinary collaboration

Not all patients were aware of the communication between the chiropractor and their GP. However, they explained that they felt it was important for the GP to be kept informed about their condition and examination results in case this turned out to be relevant to future GP contacts. Half of the patients interviewed had consulted their GP for a pain medication prescription, which was perceived as a treatment track separate from being a part of the SCCP.

One patient emphasised that it was important that only one clinician was responsible for coordinating the course of treatment, for otherwise patients risked being thrown back and forth between different care providers. The programme structure gave most patients peace of mind, and they let the chiropractor take charge of their course of treatment. Patients explained that it gave a sense of security to know that there was an opportunity for further referral (e.g., surgical assessment, MRI) if there was no positive progress in symptoms.

Some Danish patients have private health insurance covering the patient fee when seeing a chiropractor or a physiotherapist. Their health insurance will, therefore, have to approve the course of treatment prior to reimburseing costs. Patients regarded this arrangement as a barrier in the SCCP that made things unnecessarily complicated and forced them to act as a messenger between care providers. One patient described getting caught between the care delivered by SCCP on the one hand and on the other a request from their workplace and their municipal social worker for an MRI.

### Phase III: joint display

Table 3 shows a narrative side-by-side joint display of how the qualitative themes explain the survey results. Overall, thorough examinations, effective communication of diagnosis and prognosis, and coordinated care from the chiropractor were found to be the key factors in high levels of patient satisfaction. On the other hand, low levels of satisfaction were explained by unfulfilled patient expectations, absence of imaging, and inadequate patient-clinician interaction.

**Table 3:** Side-by-side joint display of quantitative and qualitative results

Quantitative results	Qualitative results		ID
*Question subject*	*Themes*	*Key quotes supporting the theme*	
**Examination**			
85% were very satisfied	Expectations regarding consultation and treatment effect	I was carefully and thoroughly examined…I think the chiropractor had a good overview after he had examined me.	6
		When she [chiropractor] told me that she wanted to see proof of the diagnosis, it made a lot of sense because the more confident she is, the more confident I become.	3
15% were less satisfied	Expectations regarding consultation and treatment effect	The first consultation lasted 18 min…it gets too hurried.	2
		I wonder why I didn’t get that MRI earlier to find out if it was actually a disc herniation… just as a precaution.	1
**Information**			
87% were very satisfied	Information about diagnosis and prognosis	It meant a lot to me that the chiropractor used a chart showing the whole body to explain how it was all related…it gives me a better insight into what is wrong.	6
		The chiropractor has been really good at explaining why it took so long…so there has been a really good dialogue.	1
13% were less satisfied	Information about diagnosis and prognosis	I had hoped that he [chiropractor] was better at explaining how much it was supposed to hurt…	2
		So, I actually get very worried when the chiropractor just says I need to move as naturally as possible… I have not been happy with the chiropractor’s information level and have not always felt that I was in good hands.	2
**Treatment effect**			
52% were very satisfied	Expectations regarding consultation and treatment effect	I had an expectation that she [chiropractor] would loosen me up so that I could walk again and get back to work and it has succeeded very well.	6
		Getting started with exercises made me get back up from my mental black hole…	4
48% were less satisfied	Expectations regarding consultation and treatment effect	I was ‘cracked’ once, then we talked, and then I was out the door. Then I thought - okay, how is this going to be better.	2
		I was in the acute phase for a long time. It is only within the last month that I feel like it is starting to pay off.	6
		If I had taken some exercises earlier, I would probably be better off now.	2
**Overall management**			
87% were very satisfied	Interdisciplinary collaboration	It’s important to me that there’s, like, a coordinator of it [the course of treatment] so I do not feel like I’m being thrown back and forth between my GP, physiotherapist and chiropractor.	3
		The chiropractor has said that, first, we will try this care package. Alternatively, if I continue to have pain every day, the chiropractor thinks we should talk to a surgeon.	5
13% were less satisfied	Interdisciplinary collaboration	I do not have a sense that there has been communication between my doctor and my chiropractor.	1
	Expectations regarding consultation and treatment effect	I just had no personal chemistry with the chiropractor. We just didn’t get on well, which I think is a good and important thing - that you understand each other.	2

Very satisfied: ≥8 on a 0–10 scale

## Discussion

The findings provided insight into patients’ satisfaction with participating in a chiropractic management programme for lumbar radiculopathy. Overall, patients had high levels of satisfaction with examination, information and the overall management, and were moderately satisfied with the treatment effect.

High levels of satisfaction were explained by thorough examinations, good communicative skills about diagnosis and prognosis, and the chiropractor’s coordination of care, whereas low levels of satisfaction were related to unmet patient expectations, lack of imaging, and poor patient-clinician interaction.

### Examination

Satisfaction was linked to the experience of a thorough examination and time spent on the consultation. This finding is in line with a systematic review [30] examining the sources of satisfaction in patients with LBP and sciatica. The review reported that patients valued what they perceived to be a thorough assessment and placed importance on care taken in constructing medical histories and on detailed examinations.

Most patients in our study perceived MRI as a part of the SCCP and explained that it brought about a better understanding of their condition and provided both patient and chiropractor with certainty and confidence by confirming the diagnosis. The two patients that did not receive an MRI, expressed disappointment in the decision. This supports studies on patients’ expectations towards the clinical examination that have reported patient approval of clinicians who ordered diagnostic imaging which they considered crucial to a thorough assessment, and the belief among some patients that imaging was more reliable than the clinical examination [30].

In the SCCP, lumbar radiculopathy is considered a clinical diagnosis, and confirmation of the diagnosis by imaging is not essential. MRI is only mandated if patients do not respond to treatment or if they develop progressive neurological deficits [15, 17]. The SCCP is supported by evidence indicating that MRI does not improve clinical outcomes and that the potential harm outweighs the potential positive effects [31], while at the same time there is no literature supporting MRI findings as a treatment effect modifier. While Danish chiropractors are authorized to refer patients for fully reimbursed MRIs as part of the collective agreement with the national healthcare system, they may find themselves in the dilemma whereby they *either* practice according to the SCCP and recommendations by clinical guidelines *or* comply with patient expectations. Approximately half of the patients with acute LBP who see their GP expect to be referred for imaging [32], and patients are unaware of potential harms caused by unnecessary imaging [33, 34]. As patient expectations strongly influence satisfaction, it requires time and good communication skills from the clinician to disseminate information to the patient regarding the pros and cons of MRI [35] that will counter unhelpful or unrealistic patient expectations.

### Information

The results showed that detailed information and explanations about diagnosis, prognosis, treatment plan and the expected duration of symptoms were highly valued by the patients, as they provided reassurance and a sense of being involved in the treatment. This finding is supported by previous research showing that patients with radiculopathy need to develop an understanding of their symptoms, how leg symptoms are related to the spine, their treatment options, and the prognosis of the disease [36].

### Treatment effect

Patients in our study expected a rapid and persistent decrease in symptoms, which unfortunately, does not match the prognosis of lumbar radiculopathy [37, 38]. Although the prognosis is considered good, the improvement happens gradually and often with fluctuating pain patterns, and it is not unusual to have milder symptoms for three months or longer [39]. In a qualitative study of patients with back-related leg pain receiving chiropractic care, patients’ satisfaction was influenced by perceived treatment effects [40]. This could partly explain why only 52% were very satisfied (≥ 8 on a 0–10 scale) with the treatment effect in our study. Furthermore, the quantitative data were collected at baseline, and it may therefore have been too early in the course of treatment to evaluate a treatment effect. However, at 8-weeks follow-up only 68% of the 182 responders (data not shown) reported high satisfaction with treatment effect, which is still lower than the other satisfaction parameters measured.

### Overall management

Satisfaction with the chiropractor’s overall coordination of care and referral to other healthcare professionals was explained by the patients’ positive experiences of coordinated care and reduced burden of responsibility. Patients with low back and leg pain are often in contact with multiple care providers either on their own initiative or as part of a multimodal treatment regime [41]. A survey of UK patients’ experiences and expectations of chiropractic care showed that two of the aspects of practice that were least met were related to the chiropractor contacting the patient’s GP and discussing referral to another healthcare provider [42]. It is possible that the SCCP, with its structured pre-scheduled follow-up sessions, scheduled standardised communication with the patient’s GP, and emphasis on a re-evaluation of the treatment plan with predefined transparent referral options may be an example of a treatment programme that could support the patient’s path through the health care system.

### Strength and limitations

We have conducted a mixed-methods study to evaluate and understand patient satisfaction with the SCCP. The use of a combination of quantitative and qualitative data not only evaluates the level of satisfaction with care in a specific patient group, but also helps us understand drivers of (dis-)satisfaction.

The survey yielded a high response rate, which gives us some confidence in the generalisability of the results. However, not all clinics invited agreed to recruit patients, and we do not know the number of patients who declined to participate, or if all relevant patients were asked to participate. It is therefore questionable whether the recruitment was truly consecutive, thus limiting the generalisability of the results.

A limitation of the study was that the satisfaction questions were developed for the current study and were not validated using an accepted validation method. The questions may be limited by a ceiling effect, as a high proportion of the patients gave the highest possible score. Although the questions were inspired by other studies of patient satisfaction [6], in which this pattern is known, it is possible that the measured satisfaction reflects the parameters of the instrument rather than patients’ true satisfaction.

The number of interviews was lower than planned, and this may limit the findings in several ways. Firstly, this may limit the generalisability of the research findings and reduce the credibility of the research results. Secondly, the results may not represent the diversity of perspectives and experiences relevant to the research question, leading to biased or narrow findings. Finally, the analysis of the data may be limited because there may not be enough data to support robust analysis or to identify patterns and themes in the data. However, as we had rich descriptions of the patients’ experiences with and perceptions of SCCP, including different views on satisfaction, we considered the qualitative data to be fairly reliable, and the combined data to be a relatively strong basis for meeting the purpose of the study.

The sequential design with the initial survey followed by interviews was chosen to evaluate the level of satisfaction and explore the issue in more depth. Ideally, we would have preferred to purposively sample both satisfied and dissatisfied patients among survey respondents to explicitly identify reasons for their level of satisfaction, but due to timing constraints, this was not possible. However, as the interviewed subjects represented both positive and negative attitudes towards the SCCP and gave detailed accounts of their experiences, we believe that the shortcomings in the patient sampling strategy have had a limited impact on the study’s findings.

Two master’s students performed the interviews as part of their master’s thesis. They received theoretical and practical training in interview techniques, yet they were novice interviewers, which may also have influenced the richness of the qualitative data and reduced information power.

### Perspectives

Based on our results, chiropractors working with SCCPs are advised to invest time in a thorough examination and focus the information provided on diagnosis, prognosis and treatment expectations. Moreover, patient-clinician interaction is essential for patient-centred care, as clinicians need to be aware of the individual patient’s pain beliefs, which should guide the delivery of information and treatment instructions. Finally, clinicians can help patients by explicitly stressing the clinician’s role in coordinating the course of treatment and making sure that patients are aware of the ongoing communication with the patient’s GP, as well as of referral options or alternative plans if the patient does not respond to treatment.

At an organisational level, it would benefit patients if the schedule allotted to follow-up sessions were less rigid. At present, the patient loses the increased reimbursement of treatment costs if the consultation is rescheduled more than +/- 2 days from the planned follow-up. This is inconvenient and expensive for the patient, and the rigid structure is supported neither by professional experience nor by any evidence.

## Conclusion

Overall, patients were satisfied with the standardised chiropractic care package for lumbar radiculopathy. From a patient’s perspective, satisfaction was linked to the chiropractor spending time on the consultation and offering a thorough examination, allowing the patient to feel in safe hands. Referral for MRI provided certainty and confidence by confirming the diagnosis. Information and guidance for patients related to variations in symptoms and expected prognosis were reassuring, and the interdisciplinary collaboration coordinated by the chiropractor (with GP, physiotherapist, and hospital referral) was highly valued.

## Electronic supplementary material

Below is the link to the electronic supplementary material.

Additional file 1: Interview guide with questions, prompts and follow-up questions.pdf.

## Data Availability

The datasets used in the current study are available from the corresponding author upon reasonable request.

## List of Abbreviations

GP: General Practitioner
GRAMMS: Good Reporting of A Mixed Methods Study
LBP: Low Back Pain
ODI: Oswestry Disability Index
NRS: Numerical Rating Scale
SD: Standard Deviation
SCCP: Standardised Chiropractic Care Package.
