# Supplementary material for: Patient expectations and levels of satisfaction in chiropractic treatment for lumbar radiculopathy. A mixed methods study
Source: Chiropr Man Therap. 2023 May 19;31:13. doi: 10.1186/s12998-023-00486-0 (PMC10197044; doi:10.1186/s12998-023-00486-0)
Supplement: Supplementary file 1 — Additional file 1: Interview guide with questions, prompts and follow-up questions.pdf. [file 12998_2023_486_MOESM1_ESM.pdf]

Additional file 1: Interview guide with questions, prompts and follow-up questions

Introduction

1. Who we are
    a. Presentation of the interviewer. Master student [name] in the last year writing my master's thesis at the University of Southern Denmark.
2. Purpose
    a. The purpose of the study is to identify which elements of the standardised chiropractic care package have an impact on the patient's attitude to the care pathway.
    b. We are interested in how you have experienced the standardised care package for lumbar radiculopathy. All answers are equally valid, and there are no right or wrong answers.
3. Time frame
    a. I expect the interview to take approx. 30 minutes.
4. I record both audio and video
    a. …so that we can return to it later and hear about your experiences again.
5. Anonymisation
    b. We treat your data/interview confidentially. Your responses will be anonymised, and it will not be possible to trace them back to you. The recording will be destroyed once the master's thesis is defended.
6. Other information
    c. If any uncertainties emerge or if something is unclear during the interview, please don't hesitate to ask.
    d. Your participation is, of course, voluntary, and you can withdraw your consent at any time.
    e. You can choose not to answer particular questions.
7. Consent
    a. Have you read the letter we sent with information about your rights and data security? Do you have any questions about it?
    b. If you consent to participate, we ask you to state your full name and acknowledge that you agree to us processing the information you provide during the interview.

Interview

8. Research questions
    a. Please tell me about the course of treatment you underwent.
        i. *Have you at any time during your time at the chiropractors heard the term "standardised care package "?*

    ii. *What thoughts went through your mind when the chiropractor mentioned a "standardised care package"?*
    iii. *What is your understanding of the "standardised care package" at the chiropractor?*
    iv. *What were your expectations of the chiropractor? Were they met?*
    v. *Was there anything in the process that made you wonder?*

b. What was your experience regarding the examination you received at the chiropractor?
    i. *Do you think the chiropractor had a good overview of your situation, after his/her examination of you?*
    ii. *Did you have expectations for your examination that were not met by your chiropractor?*
        - *Were you referred for MRI, and what did you think of the decision?*

c. What do you think about the information you received from the chiropractor?
    i. *Was there information you were particularly pleased to receive?*
    ii. *Was there any specific information you think was missing?*
        - *Where did you then get this information?*
    iii. *Did you get any written information? A booklet or anything like that?*
    iv. *Were the questions you asked answered?*
    v. *Were you informed about why you were included in the standardised care package?*

d. Please tell me about the treatment you received at the chiropractor.
    i. *What did the treatment consist of?*
    ii. *Did it live up to your expectations? Effect of treatment?*
    iii. *Did you feel that you were treated as an individual or as a person with a medical problem?*

e. What are your thoughts about the structure of the course of treatment?
    i. *Did you have an influence over what was to happen in the process?*
        - *How did it express itself?*
    ii. *What do you think about the pre-scheduled follow-ups in the programme?*
        - *Reassuring? Annoying? Should there be more/fewer?*
    iii. *Have you seen your GP about this issue?*
        - *What treatment was then provided?*
        - *Did you find that your GP and your chiropractor cooperated during your course of treatment?*
    iv. *Did you feel in safe hands with your chiropractor?*
        - *Did you talk about the need for further examination or referral, for example, for a scan or to a hospital?*

f. Was there anything in the process that you were dissatisfied with? Perhaps based on the previously mentioned topics.

g. Was there anything that could have improved your treatment or could improve treatment for others with a similar condition?
    a. Please describe

h. Was there anything you were particularly pleased with?
    a. Please describe

i. Would you accept being enrolled in a standardised care package again if you got another slipped disc tomorrow?
    a. *Would you recommend such a package to others (friends/family)?*

j. Do you have any further thoughts about the course of treatment that you think are important to tell me?

Informant information

9. Personal information about you

   a. How old are you?
   b. What is your longest completed course of higher/further education?
